# ﻿Three new species of the genus *Bambusiphaga* Huang & Ding, 1979 (Hemiptera, Fulgoroidea, Delphacidae, Tropidocephalini) from China, with an updated checklist and key to species

**DOI:** 10.3897/zookeys.1217.125780

**Published:** 2024-11-08

**Authors:** Sha-Sha Lv, Hong-Xing Li, Lin Yang, Yu-Bo Zhang, Xiang-Sheng Chen

**Affiliations:** 1 Institute of Entomology, Guizhou University, Guiyang, Guizhou, 550025, China; 2 The Provincial Special Key Laboratory for Development and Utilization of Insect Resources of Guizhou, Guizhou University, Guiyang, Guizhou, 550025, China; 3 Guizhou Light Industry Technical College, Guiyang, Guizhou, 561113, China; 4 Anshun University, College Agriculture, Anshun, Guizhou, 561000, China

**Keywords:** Bamboo pests, Fulgoroidea, identification key, Oriental region, taxonomy, Tropidocephalini

## Abstract

In this study, three new bamboo-feeding species of the genus *Bambusiphaga* Huang & Ding, 1979 (Hemiptera, Fulgoroidea, Delphacidae, Tropidocephalini), *B.caudospina* Lv, Li & Chen, **sp. nov.**, *B.laterospina* Lv, Li & Chen, **sp. nov.** and *B.striola* Lv, Li & Chen, **sp. nov.** from Southwest China, are described and illustrated, bringing the total number of species in the genus to 34. An updated identification key and checklist to all known species of *Bambusiphaga* are provided.

## ﻿Introduction

The bamboo-feeding genus *Bambusiphaga* Huang & Ding, 1979 (Delphacidae, Delphacinae, Tropidocephalini) was established by Huang and Ding for six species feeding on *Neosinocalamusaffinis* (Rendle) and *Phyllostachys* sp. (Poales, Poaceae), with *B.nigropunctata* Huang & Ding, 1979 as the type species from Sichuan Province, China (Huang et al. 1979). Then Kuoh et al. (1980) described two new species, *B.fascia* Huang & Tian, 1980 and *B.nigromarginata* Huang & Tian, 1980. Ding (1982) and Ding and Hu (1982) both added a new species in 1982. Asche (1983) described a new species from East Himalaya, *B.lynchi* Asche, 1983, and transferred the following two species from the genus *Columbisoga* Muir, 1921 into *Bambusiphaga*: *B.taiwanensis* (Muir, 1917) and *B.singaporensis* (Muir, 1919). Ding et al. (1986) and Yang and Yang (1986) described *B.jinghongensis* Ding & Hu, 1986 and *B.membranacea* Yang & Yang, 1986 from Yunnan and Taiwan Provinces in China, respectively. Chen et al. (2000) described two new species, *B.maculata* Chen & Li, 2000 and *B.wangmoensis* Chen & Li, 2000, attacking bamboo from Guizhou Province in China. Qin et al. (2006) transferred *Malaxabakeri* Muir, 1919 into the genus. Chen and Liang (2007) revised *Bambusiphaga* and added two species, *B.maolanensis* Chen & Liang, 2007 and *B.pianmaensis* Chen & Liang, 2007. Since then, 11 species have been added to the genus (Hou and Chen 2010; Yang and Chen 2011; Qin et al. 2012; Li et al. 2018, 2023; Ramya and Meshram 2019). Until now, 31 species have been recorded in the genus, which is widely distributed in the Oriental region, with 28 species in China, one in the Philippines, two in Singapore, two in Malaysia, one in the North-Eastern Himalayas, and one in India (Yang and Chen 2011; Qin et al. 2012; Li et al. 2018, 2023; Ramya and Meshram 2019; Bourgoin 2024).

Herein, three new species of the genus, *B.caudospina* sp. nov., *B.laterospina* sp. nov. and *B.striola* sp. nov. from Southwest China, are described and illustrated. As a result, the number of *Bambusiphaga* species has increased to 34, with 31 recorded from China.

## ﻿Materials and methods

The external morphology terminologies are as follows: male genitalia follows Yang and Yang (1986) and Bourgoin (1987), and wing venation follows Bourgoin et al. (2015). Dry male specimens were used for the descriptions and illustrations. Body measurements are from the apex of the vertex to the tip of the forewing. All measurements are in millimeters (mm). External morphology and drawings were done under the Leica MZ 12.5 stereomicroscope. Color pictures for adult habitus were obtained by the KEYENCE VHX-6000 system. The photographs and illustrations were scanned with a CanoScan LiDE 200 and imported into Adobe Photoshop 6.0 for labeling and plate composition. The dissected male genitalia are preserved in glycerin jelly in small plastic tubes pinned together with the specimens.

The type specimens examined are deposited in the Institute of Entomology, Guizhou University, Guiyang, Guizhou Province, China (IEGU).

## ﻿Taxonomy

### ﻿ Bambusiphaga

Taxon classificationAnimaliaHemipteraDelphacidae

Huang & Ding, 1979

1F49478A-B37D-50B9-8247-9CE95E170C8F

Bambusiphaga Huang & Ding, 1979: 170; Asche 1983: 211; Ding and Tian 1983 (in Kuoh et al. 1983): 49; Yang and Yang 1986: 37; Wang and Ding 1996: 22; Ding et al. 1999: 441; Ding 2006: 126; Chen and Liang 2007: 504; Hou and Chen 2010: 392; Yang and Chen 2011: 51; Li et al. 2018: 84, 2023: 143.

#### Type species.

*Bambusiphaganigropunctata* Huang & Ding, 1979, original designation.

#### Diagnosis.

For the diagnosis of *Bambusiphaga* see Chen and Liang (2007: 504) and Li et al. (2023: 143).

#### Host plants.

Bamboo (Bambusoideae).

#### Distribution.

China, India, Malaysia, North-Eastern Himalayas, Philippines, Singapore.

##### ﻿Checklist and distributions of species of *Bambusiphaga* Huang & Ding, 1979

*B.angulosa* Li, Chen & Yang, 2023; China (Yunnan Province).

*B.bakeri* (Muir, 1919); China (Guangdong, Guizhou, Hainan, Shaanxi, Taiwan Provinces), Philippines (Luzón, Laguna), Malasia (Peninsula), Singapore.

*B.basifusca* Hou & Chen, 2010; China (Hainan Province).

*B.caudospina* Lv, Li & Chen, sp. nov.; China (Guizhou Province).

*B.citricolorata* Huang & Tian, 1979; China (Guizhou, Yunnan Provinces).

*B.fascia* Huang & Tian, 1980; China (Anhui, Chongqing, Gansu, Guizhou, Jiangsu, Sichuan, Taiwan, Zhejiang Provinces).

*B.furca* Huang & Ding, 1979; China (Fujian, Guizhou, Taiwan, Yunnan Provinces).

*B.hainanensis* Hou & Chen, 2010; China (Hainan Province).

*B.huangi* Ding & Hu, 1982; China (Yunnan Province).

*B.jinghongensis* Ding & Hu, 1986; China (Yunnan Province).

*B.kunmingensis* Yang & Chen, 2011; China (Yunnan Province).

*B.lacticolorata* Huang & Ding, 1979; China (Guizhou, Jiangsu, Zhejiang Provinces).

*B.laterospina* Lv, Li & Chen, sp. nov.; China (Yunnan Province).

*B.luodianensis* Ding, 1982; China (Guangxi, Guizhou, Hainan Provinces).

*B.lynchi* Asche, 1983; North-Eastern Himalaya.

*B.maculata* Chen & Li, 2000; China (Guizhou, Henan Provinces).

*B.maolanensis* Chen & Liang, 2007; China (Guizhou Province).

*B.membranacea* Yang & Yang, 1986; China (Guizhou, Taiwan Provinces).

*B.mirostylis* Huang & Ding, 1979; China (Yunnan Province).

*B.nigrigena* Li, Chen & Yang, 2023; China (Yunnan Province).

*B.nigromarginata* Huang & Tian, 1980; China (Jiangsu Province).

*B.nigropunctata* Huang & Ding, 1979; China (Guangxi, Guizhou, Gansu, Hainan, Shaanxi, Sichuan Provinces).

*B.parvula* Li, Chen & Yang, 2023; China (Yunnan Province).

*B.pianmaensis* Chen & Liang, 2007; China (Yunnan Province).

*B.similis* Huang & Tian, 1979; China (Yunnan Province).

*B.singaporensis* (Muir, 1919); Malasia (Peninsula), Singapore.

*B.striola* Lv, Li & Chen, sp. nov.; China (Tibet Province).

*B.taibaishana* Qin, Liu & Lin, 2012; China (Shaanxi Province).

*B.taiwanensis* (Muir, 1917); China (Fujian, Guizhou, Taiwan Provinces).

*B.unispina* Ramya & Meshram, 2019; India (Himachal Pradesh).

*B.ventroprocessa* Li, Yang & Chen, 2018; China (Hainan Province).

*B.wangmoensis* Chen & Li, 2000; China (Guizhou Province).

*B.yangi* Yang & Chen, 2011; China (Yunnan Province).

*B.yingjiangensis* Li, Yang & Chen, 2018; China (Yunnan Province).

### ﻿Key to species of *Bambusiphaga* Huang & Ding, 1979

Modified from Li et al. 2018.

**Table:** 

1	Vertex dark brown or with dark brown spots	**2**
–	Vertex light, without dark brown spots	**4**
2	Vertex yellowish-brown, middle part of basal compartment with a black oval spot; anal segment without a process; pygofer without medioventral processes (Huang et al. 1979: figs 2, 4)	***B.nigropunctata* Huang & Ding, 1979**
–	Vertex brownish-black, middle part of basal compartment without a black oval spot; anal segment with a thick and long process; pygofer with a pair of medioventral processes	**3**
3	Anal segment with process distinctly divided into 2 processes at apex; apical part of aedeagus without two unciform processes (Chen and Liang 2007: figs 46, 53)	***B.pianmaensis* Chen & Liang, 2007**
–	Anal segment with process distinctly divided into 3 processes at apex (Fig. 6C, E); apical part of aedeagus with two unciform processes (Fig. 6J)	***B.striola* Lv, Li & Chen, sp. nov.**
4	Mesonotum with dark brown markings	**5**
–	Mesonotum without dark brown markings	**17**
5	Lateral areas of pronotum with dark brown markings	**6**
–	Lateral areas of pronotum without dark brown markings	**15**
6	Forewings with a large irregular pale brown stripe along transverse vein, hence bending along posterior margin to apex (Li et al. 2018: fig. 8)	***B.yingjiangensis* Li, Yang & Chen, 2018**
–	Forewings with basal ⅓ black or with black markings at basal half	**7**
7	Forewings with basal ⅓ black	**8**
–	Forewings with large black markings at base	**12**
8	Anal segment without a process on ventral margin (Yang and Chen 2011: fig. 6)	***B.kunmingensis* Chen & Yang, 2011**
–	Anal segment with a very long process on ventral margin	**9**
9	Anal segment with a spiny process at right lateroapical angle (Hou and Chen 2010: fig. 14)	***B.basifusca* Hou & Chen, 2010**
–	Anal segment with a spiny process at left lateroapical angle	**10**
10	Pygofer without medioventral process; apical half of aedeagus without two processes (Ding 2006: fig. 54c, f)	***B.fascia* Huang & Tian, 1980**
–	Pygofer with medioventral process; apical half of aedeagus with two processes	**11**
11	Pygofer with a short medioventral process, without lateroventral process; genital styles without process at middle part (Qin et al. 2012: figs 12, 16, 17)	***B.taibaishana* Qin, 2012**
–	Pygofer with a pair long medioventral and a lateroventral processes (Fig. 4C, D); inner margin of genital styles with a toothed process at middle part (Fig. 4C, G)	***B.laterospina* Lv, Li & Chen, sp. nov.**
12	Forewings with two large black markings at base; anal segment without process on ventral margin (Li et al. 2018: figs 29, 31)	***B.ventroprocessa* Li, Yang & Chen, 2018**
–	Forewings with one large black marking at base; anal segment with a long process on ventral margin	**13**
13	Anal segment with a long ventral process medially; pygofer without medioventral process (Li et al. 2023: fig. 6C, F)	***B.nigrigena* Li, Chen & Yang, 2023**
–	Anal segment with a long ventral process at left lateroapical angle; pygofer with medioventral process	**14**
14	Genital styles branched at apical part; medioventral process forked near base (Chen et al. 2000: figs 4, 7, 8)	***B.maculata* Chen & Li, 2000**
–	Genital styles unbranched at apical part (Fig. 2G–I); medioventral process forked near apical 1/2 (Fig. 2C, F)	***B.caudospina* Lv, Li & Chen, sp. nov.**
15	Mesonotum without black marking in middle (Ramya and Meshram 2019: fig. 3)	***B.unispina* Ramya & Meshram, 2019**
–	Mesonotum with black markings in middle	**16**
16	Forewings somewhat reddish-orange, costal margin dark brown; genital styles relatively broad and short (Kuoh et al. 1980: fig. 8c, f)	***B.nigromarginata* Huang & Tian, 1980**
–	Forewings somewhat yellowish-brown, costal margin yellowish-brown; genital styles relatively slender (Yang and Yang 1986: fig. 20c, e)	***B.taiwanensis* (Muir, 1917)**
17	Anal segment with a process on ventral margin	**18**
–	Anal segment without a process on ventral margin	**22**
18	Pygofer with medioventral process (Ding 2006: fig. 62c)	***B.bakeri* (Muir, 1919)**
–	Pygofer without medioventral process	**19**
19	Anal segment with the process long and extends to ventral margin of pygofer	**20**
–	Anal segment with the process short	**21**
20	Body small, about 3.5–3.6 mm; genital styles with a process at base, apex rounded (Ding et al. 1986: figs 1(5–6))	***B.jinghongensis* Ding & Hu, 1986**
–	Body slightly larger, about 4.3 mm; genital styles without a process at base, apex forked (Huang et al. 1979: fig. 18)	***B.mirostylis* Huang & Ding, 1979**
21	Tegula dark brown at apical ½; hind margin of pygofer produced at an acute angle medially; genital styles slender; aedeagus without phallobase (Ding and Hu 1982: figs 1–4)	***B.huangi* Ding & Hu, 1982**
–	Tegula yellowish-brown; hind margin of pygofer not produced medially; genital styles broad and short; aedeagus with developed phallobase (Chen and Li 2000: figs 11, 13, 15–16)	***B.wangmoensis* Chen & Li, 2000**
22	Ventral margin of pygofer with a spine	**23**
–	Ventral margin of pygofer without a spine	**25**
23	Caudal side of genital styles with an inversed spine near apex; aedeagus with three spines subapically (Yang and Chen 2011: figs 20–22)	***B.yangi* Chen & Yang, 2011**
–	Caudal side of genital styles with an angular or tooth-like process near apex; aedeagus without spines subapically	**24**
24	Genital styles asymmetrical; aedeagus with an inversed process on right side near apical ⅓ (Muir 1919: fig. 8)	***B.singaporensis* (Muir, 1919)**
–	Genital styles symmetrical; aedeagus without any processes (Ding 1982: figs 3, 5)	***B.luodianensis* Ding, 1982**
25	Basal part of genital styles with a fingerlike process	**26**
–	Basal part of genital styles without a fingerlike process	**27**
26	Genital styles with a fingerlike process subapically; aedeagus curved medially (Chen and Liang 2007: figs 20–22)	***B.maolanensis* Chen & Liang, 2007**
–	Genital styles with a lamellate process subapically; aedeagus almost straight (Hou and Chen 2010: figs 9–10)	***B.hainanensis* Hou & Chen, 2010**
27	Genital styles forked apically	**28**
–	Genital styles unforked apically	**30**
28	Frons longer at midline than maximum width, about 2.0: 1; basocaudal part of genital styles in profile produced at a right angle (Yang and Yang 1986: fig. 22b, h)	***B.membranacea* Yang & Yang, 1986**
–	Frons longer at midline than maximum width, about 2.5: 1; basocaudal part of genital styles in profile not produced at a right angle	**29**
29	Middle part of genital styles granulate (Huang et al. 1979: figs 8–11)	***B.furca* Huang & Ding, 1979**
–	Middle part of genital styles not granulate (Asche 1983: fig. 4)	***B.lynchi* Asche, 1983**
30	Ventral margin of anal segment incised medially; genital styles short (Huang et al. 1979: fig. 20)	***B.lacticolorata* Huang & Ding, 1979**
–	Ventral margin of anal segment not incised medially; genital styles slender	**31**
31	Genital styles with a spinous process near apex	**32**
–	Genital styles without a spinous process near apex	**33**
32	Aedeagus with some small teeth near apex, not forked at apex (Li et al. 2023: fig. 2H–I)	***B.parvula* Li, Chen & Yang, 2023**
–	Aedeagus without small teeth near apex, forked at apex (Li et al. 2023: fig. 4H–I)	***B.angulosa* Li, Chen & Yang, 2023**
33	Apex of vertex obviously broadened, frons widest at base; apex of genital styles without small teeth; aedeagus short and stout (Huang et al. 1979: fig. 17)	***B.similis* Huang & Tian, 1979**
–	Apex of vertex unbroadened, frons widest at apex; apex of genital styles with several small teeth; aedeagus relatively long (Huang et al. 1979: figs 13–15)	***B.citricolorata* Huang & Tian, 1979**

### ﻿ Bambusiphaga caudospina

Taxon classificationAnimaliaHemipteraDelphacidae

Lv, Li & Chen sp. nov.

68A0E358-7F51-55DF-A57A-1CD7C28171F4

https://zoobank.org/374EE33D-FD7B-4ED4-8736-4E84E8D058A3

Figs 1 , 2

#### Type material.

***Holotype***: China • ♂: Guizhou Province, Weining County, Xueshan Town; 27°4'N, 104°7'E; sweeping, 4 August 2023; Hong-Xing Li leg.; IEGU. ***Paratypes***: China • 8 ♂♂, 12 ♀♀; Guizhou Province, Weining County, Xueshan Town; 27°4'N, 104°7'E; sweeping, 4 August 2023; Hong-Xing Li and Jie Wang leg.; IEGU.

#### Diagnosis.

**Figure 1.**
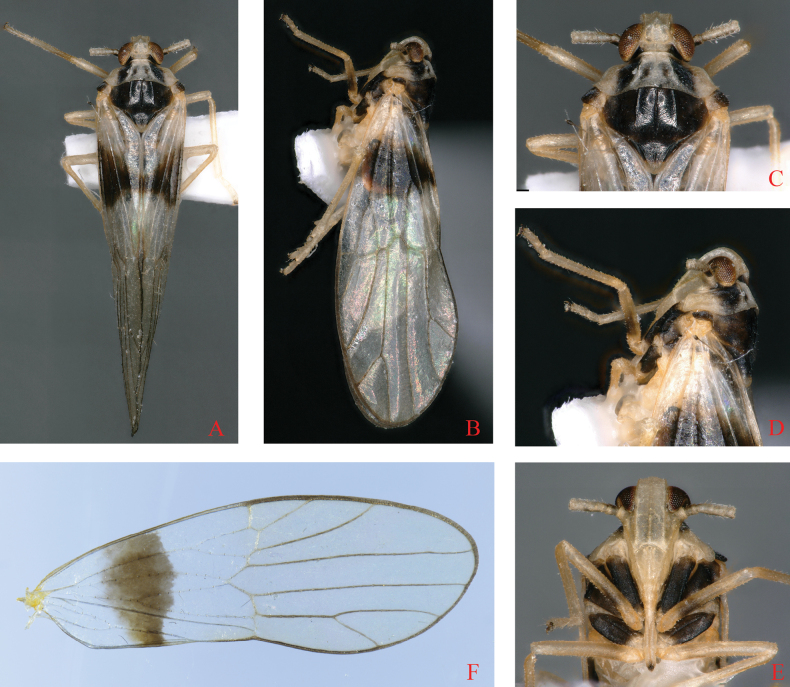
*Bambusiphagacaudospina* Lv, Li & Chen, sp. nov., male **A** habitus, dorsal view **B** habitus, lateral view **C** head and thorax, dorsal view **D** head and thorax, lateral view **E** frons, ventral view **F** forewing.

**Figure 2.**
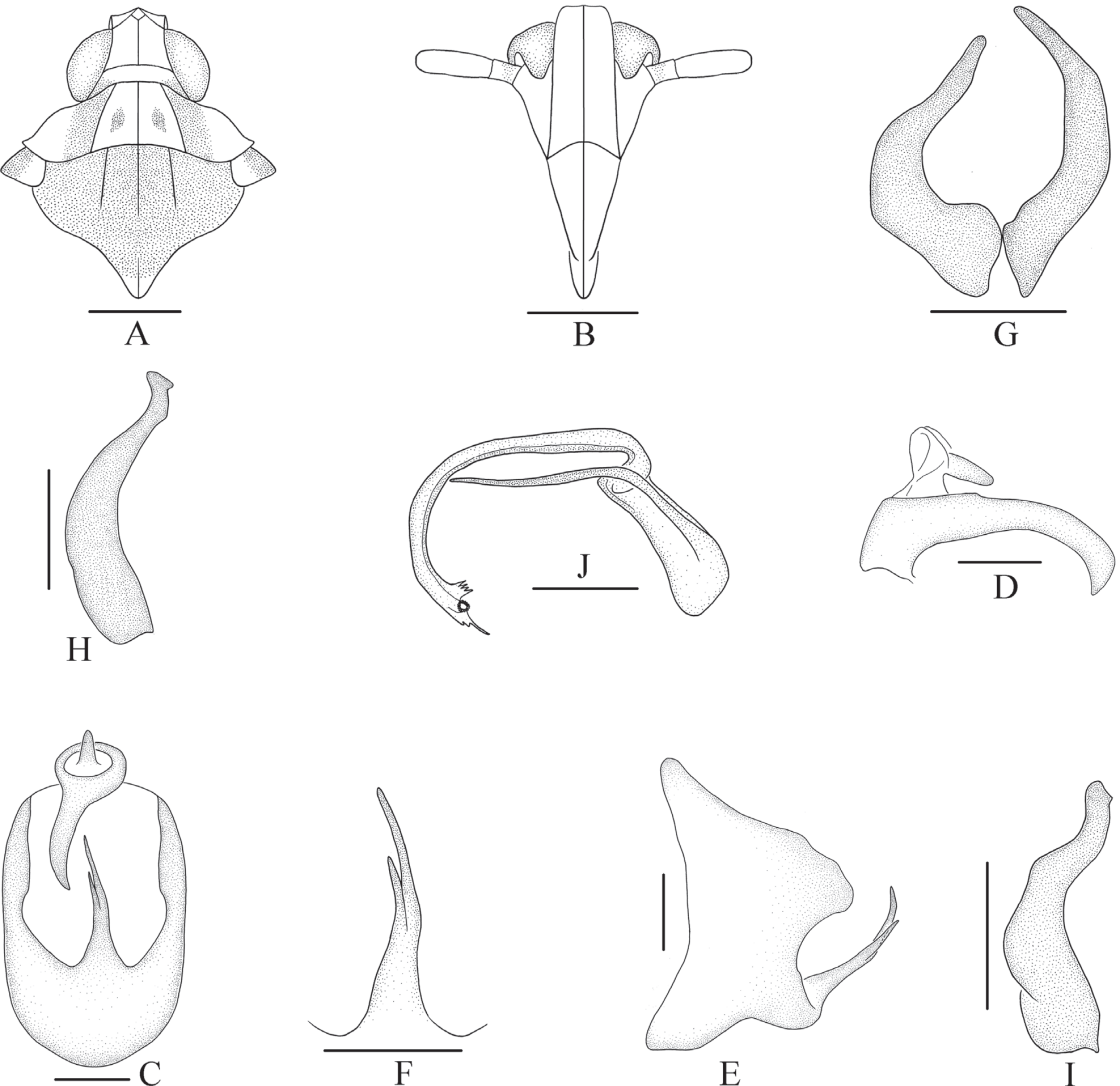
*Bambusiphagacaudospina* Lv, Li & Chen, sp. nov., male **A** head and thorax, dorsal view **B** frons, ventral view **C** male genitalia, posterior view **D** anal segment, lateral view **E** pygofer, lateral view **F** medioventral process of pygofer, posterior view **G** genital style, posterior view **H** left genital style, lateral view **I** right genital style, lateral view **J** aedeagus, lateral view. Scale bars: 0.5 mm (**A, B**); 0.2 mm (**C–J**).

The salient features of the new species include: vertex (Fig. 1A, C) light, without dark brown spots; lateral areas of pronotum (Fig. 1A, C) with dark brown markings; mesonotum (Fig. 1A, C) with dark brown markings; forewings (Fig. 1F) with one large black marking at basal 1/3; medioventral process of pygofer (Fig. 2C, F) forked near apical 1/2; dorsolateral margin of aedeagus (Fig. 2J) with three spinous processes at apical part, ventrolateral margin with four spinous processes of similar length. This species is similar to *B.maculata* Chen & Li, 2000, but differs from the latter in: (1) forewings MP_1+2_ fully commingled (forewings MP_1+2_ commingled at base in *B.maculata*); (2) apical part of genital styles not forked (apical part of genital styles forked in *B.maculata*); and (3) apical part of aedeagus with some spinous processes on both sides (apical part of aedeagus with some spinous processes on only one side in *B.maculata*).

#### Description.

***Measurements*.** Total length: male 4.6–4.9 mm (*N* = 9), female 5.0–5.3 mm (*N* = 12).

***Coloration*.** General color yellowish-white to black (Fig. 1A–F). Vertex and frons yellowish-white, rostrum blackish-brown at apex. First segment of antennae yellowish-white at dorsal and ventral sides, lateral sides dark brown, second segment yellowish-white. Eyes reddish-brown. Pronotum yellowish-white, outer sides of lateral carinae with black broad stripes, inner sides with two dark brown round spots. Mesonotum black, lateral margins yellowish-brown, apex of scutellum opalescent. Outer part of tegula black brown, inner part yellowish-white. Forewings milky-white, hyaline, basal 1/3 with a dark brown, wide transverse marking. Legs yellowish-white, except coxae of fore and median legs dark brown.

***Head and thorax*.** Vertex (Figs 1C, 2A) shorter in middle line than wide at base (1: 1.11), width at apex narrower than at base (1: 1.19), middle part of anterior margin convex, lateral margins widened towards the end, lateral and submedian carinae distinct, Y-shaped carina indistinct. Frons (Figs 1E, 2B) longer in middle line than wide at widest portion (about 2.12: 1), widest at apex, median carina simple. Base of postclypeus (Figs 1E, 2B) as wide as apex of frons. Antennae (Figs 1E, 2B) with first segment longer than wide, shorter than second segment (1: 3.33). Pronotum (Figs 1C, 2A) longer than vertex in midline (1.14: 1). Mesonotum (Figs 1C, 2A) longer than 1.17 times pronotum and vertex combined. Forewings (Fig. 1F) slender, longer than maximal width (2.81: 1).

***Male genitalia*.** Pygofer ventral margin distinctly longer than dorsal margin in lateral view (Fig. 2E), in posterior view (Fig. 2C) with opening longer than wide, ventral margin with long medioventral process, forked near apical 1/2, right branch longer than left one. Anal segment (Fig. 2C, D) ring-like, with a thick and long anal process at left lateroapical angle, taper the end and bend to the right. Genital styles (Fig. 2G–I) moderately long, wide at base, tapering at the end, in posterior view asymmetrical, in lateral view apex truncated. Aedeagus (Fig. 2J) with phallobase, phallus tubular, basal part thick, apical part thin, ventrally curved at basal 1/3; dorsolateral margin of apical part with three spinous processes, inner one much longer than the other two, ventrolateral margin with four spinous processes of similar length; gonopore located at apex of phallus, node-like; phallobase slender and long, arched medially.

#### Host plant.

Bamboo.

#### Distribution.

China (Guizhou Province).

#### Etymology.

The species name is a combination of the Latin word “*caudo*-” and “*spina*”, referring to apical part of aedeagus with spinous processes.

### ﻿ Bambusiphaga laterospina

Taxon classificationAnimaliaHemipteraDelphacidae

Lv, Li & Chen sp. nov.

81CAF63D-12F2-5A6D-83C6-EE027AB77ED4

https://zoobank.org/077D3B46-9854-4474-B64E-C8DE09459674

Figs 3 , 4

#### Type material.

***Holotype***: China • ♂; Yunnan Province, Lushui City; 25°50'N, 98°54'E; sweeping, 8 August 2023; Yong-Jin Sui and Feng-E Li leg.; IEGU. ***Paratypes***: China • 13 ♂♂, 6 ♀♀; same collection data as for holotype; IEGU.

#### Diagnosis.

**Figure 3.**
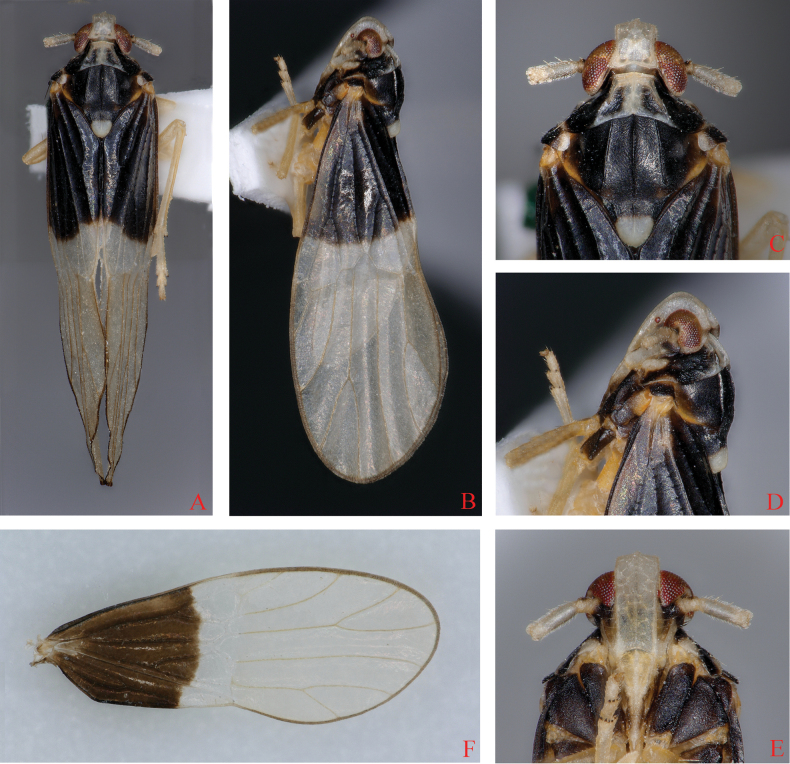
*Bambusiphagalaterospina* Lv, Li & Chen, sp. nov., male **A** habitus, dorsal view **B** habitus, lateral view **C** head and thorax, dorsal view **D** head and thorax, lateral view **E** frons, ventral view **F** forewing.

**Figure 4.**
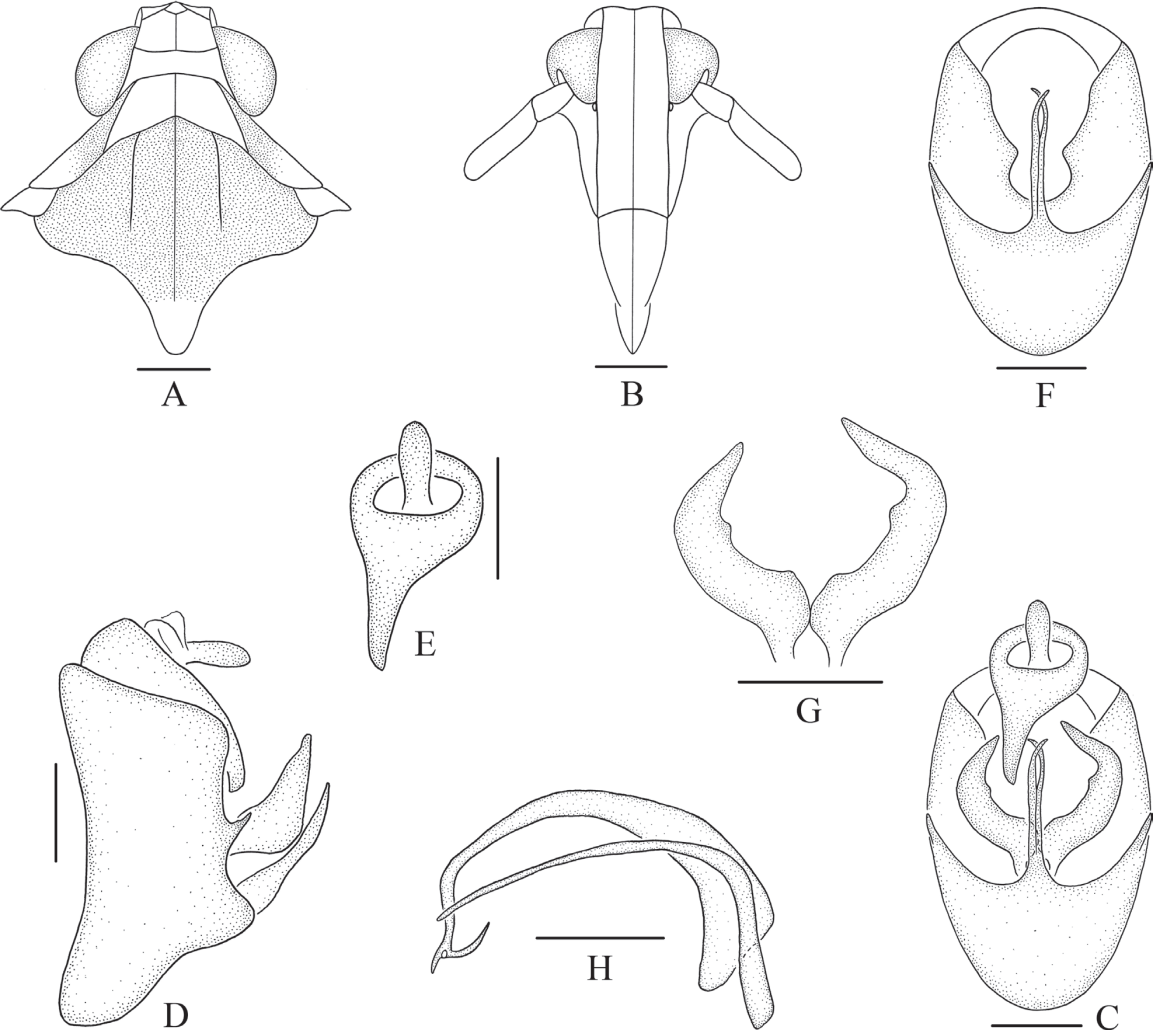
*Bambusiphagalaterospina* Lv, Li & Chen, sp. nov., male **A** head and thorax, dorsal view **B** frons, ventral view **C** male genitalia, posterior view **D** male genitalia, lateral view **E** anal segment, posterior view **F** pygofer, posterior view **G** genital style, posterior view **H** aedeagus, lateral view. Scale bars: 0.5 mm (**A, B**); 0.2 mm (**C–H**).

The salient features of the new species include: vertex (Fig. 3A, C) light, without dark brown spots; lateral areas of pronotum (Fig. 3A, C) with dark brown markings; mesonotum (Fig. 3A, C) with dark brown markings; forewings (Fig. 3F) with basal ⅓ black; pygofer (Fig. 4C, D) with long medioventral and lateroventral processes; inner margin of genital styles (Fig. 4C, G) with a toothed process at middle part; apical part of aedeagus (Fig. 4H) with two slender spinous processes. This species is similar to *B.taibaishana* Qin, 2012, but differs from the latter in: (1) outer part of tegula black brown, inner part yellowish-white (tegula black brown in *B.taibaishana*); (2) pygofer with a pair long medioventral and a lateroventral processes (pygofer with a short medioventral process, without lateroventral process in *B.taibaishana*); and (3) inner margin of genital styles with a toothed process at middle part (genital styles without process at middle part in *B.taibaishana*).

#### Description.

***Measurements*.** Total length: male 4.7–4.9 mm (*N* = 14), female 4.9–5.1 mm (*N* = 6).

***Coloration*.** General color yellowish-white to black (Fig. 3A–F). Vertex, frons and clypeus yellowish-white. Genae dark brown at base, yellowish-white at apex. Eyes and ocelli reddish-brown. First segment of antennae yellowish-white except for dorsal side dark brown. Pronotum with lateral carinae yellowish-white at inner side, with two dark brown triangular spots, outer side black. Mesonotum black, lateral margins yellow, apex of scutellum yellowish-white. Outer part of tegula black brown, inner part yellowish-white. Forewings pale yellowish-white, hyaline, blackish-brown at basal 1/3. Legs yellowish-white, except coxae of fore and median legs dark brown.

***Head and thorax*.** Vertex (Figs 3C, 4A) shorter in middle line than wide at base (0.90: 1), width at apex narrower than at base (0.82: 1), middle part of anterior margin convex, lateral margins widened towards the end, lateral and submedian carinae distinct, Y-shaped carina distinct. Frons (Figs 3E, 4B) longer in middle line than wide at widest portion (about 2.80: 1), widest at apex, median carina simple. Base of postclypeus (Figs 3E, 4B) as wide as apex of frons. Antennae (Figs 3E, 4B) with first segment longer than wide, shorter than second segment (1: 2.0). Pronotum (Figs 3C, 4A) equal in length to vertex in midline (1.14: 1). Mesonotum (Figs 3C, 4A) longer than 2.08 times pronotum and vertex combined. Forewings (Fig. 3F) slender, longer than maximal width (2.57: 1).

***Male genitalia*.** Pygofer ventral margin longer than dorsal margin in lateral view (Fig. 4D), in posterior view (Fig. 4C) with opening longer than wide, ventral margin with long medioventral process, forked near apical 1/2, lateral margins each with a lateroventral process. Anal segment (Fig. 4C–E) ring-like, with a thick and long anal process at left lateroapical angle, taper the end. Genital styles (Fig. 4C, D, G) moderately long, hogged, wide at base, tapering at the end, inner margin with a toothed process near the middle. Aedeagus (Fig. 4H) with phallobase, phallus tubular, curved ventrally, basal part thick, apical part thin, apical part with two slender spinous processes; gonopore located at apex of phallus; phallobase slender and long, arched near basal 1/3.

#### Host plant.

Bamboo.

#### Distribution.

China (Yunnan Province).

#### Etymology.

The species name is a combination of the Latin word “*latero*-” and “*spina*”, referring to lateral margins of pygofer, each with a lateroventral process.

### ﻿ Bambusiphaga striola

Taxon classificationAnimaliaHemipteraDelphacidae

Lv, Li & Chen sp. nov.

110713BA-358F-5C26-AF2A-FAE47751398F

https://zoobank.org/4C51A91E-4402-4A2A-B7C2-BD3564D53DA3

Figs 5 , 6

#### Type material.

***Holotype***: China • ♂; Tibet Province, Milin County, Milin Town; 29°13'N, 94°13'E; sweeping, 22 August 2022; Yong-Jin Sui leg.; IEGU. ***Paratypes***: China • 13 ♂♂, 15 ♀♀; same collection data as for holotype; IEGU.

#### Diagnosis.

**Figure 5.**
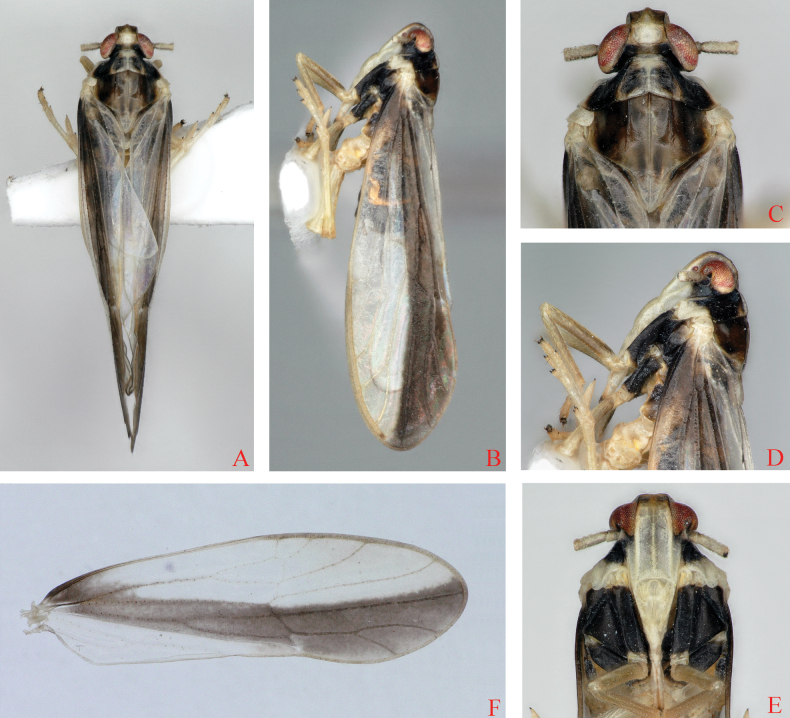
*Bambusiphagastriola* Lv, Li & Chen, sp. nov., male **A** habitus, dorsal view **B** habitus, lateral view **C** head and thorax, dorsal view **D** head and thorax, lateral view **E** frons, ventral view **F** forewing.

**Figure 6.**
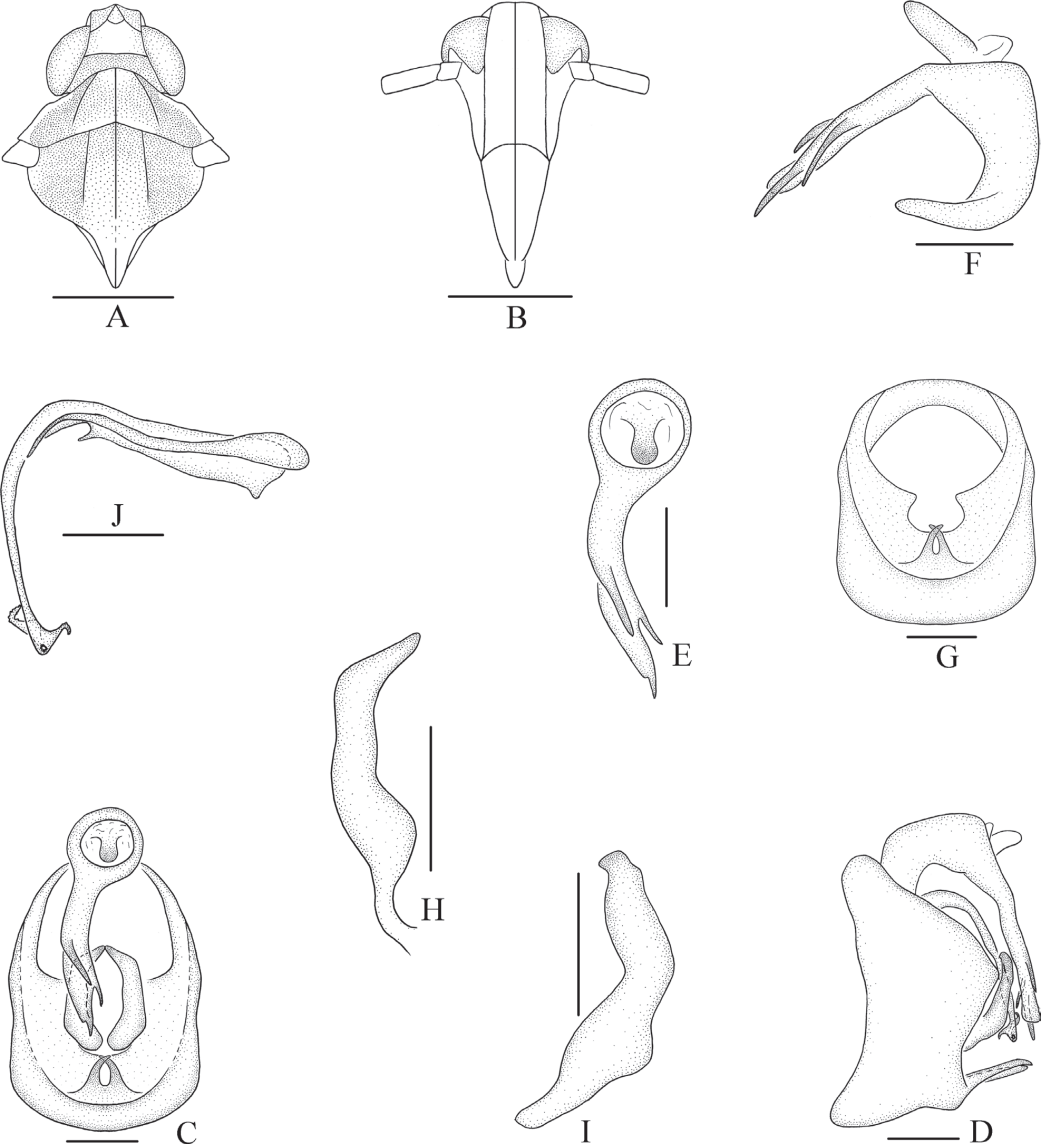
*Bambusiphagastriola* Lv, Li & Chen, sp. nov., male **A** head and thorax, dorsal view **B** frons, ventral view **C** male genitalia, posterior view **D** male genitalia, lateral view **E** anal segment, posterior view **F** anal segment, lateral view **G** pygofer, posterior view **H** genital style, posterior view **I** genital style, lateral view **J** aedeagus, lateral view. Scale bars: 0.5 mm (**A, B**); 0.2 mm (**C–J**).

The salient features of the new species include: vertex (Fig. 5A, C) brownish-black, basal compartment milky-white; tegula (Fig. 5A, C) milky-white; forewings (Fig. 5F) with a dark brown longitudinal band from anterior margin of basal part to posterior margin of apical part along the CuP and MP; pygofer (Fig. 6C, G) with a pair of medioventral processes; anal segment (Fig. 6C, E) with the process distinctly divided into 3 processes at apex; apical part of aedeagus (Fig. 6J) with two unciform processes, basal and middle parts each with a dentate processes. This species is similar to *B.pianmaensis* Chen & Liang, 2007, but differs from the latter in: (1) posterior margin of pronotum milky-white at middle part (posterior margin of pronotum blackish-brown at middle part in *B.pianmaensis*); (2) apical part of anal segment divided into 3 processes at apex (apical part of anal segment divided into 2 processes at apex in *B.pianmaensis*); and (3) basal and middle parts of aedeagus each with a dentate process (basal and middle parts of aedeagus without a dentate process in *B.pianmaensis*).

#### Description.

***Measurements*.** Total length: male 4.8–5.0 mm (*N* = 14), female 5.3–5.6 mm (*N* = 15).

***Coloration*.** General color milky-white to dark brown (Fig. 5A–F). Vertex blackish-brown, basal compartment milky-white. Frons milky-white, basal part with two blackish-brown spots. Clypeus milky-white. Genae basal 1/4 dark brown, rest milky-white. Antennae light brown. Pronotum with lateral carinae yellowish-white at inner side, with two dark brown spots, outer side black, lateral margins yellowish-white. Mesonotum pale yellowish-brown, lateral sides with two blackish-brown spots, lateral margins yellow, apex of scutellum yellowish-white. Tegula milky-white. Forewings translucent, with a dark brown longitudinal band from anterior margin of basal part to posterior margin of apical part along CuP and MP. Legs yellowish-white, except coxae of fore and median legs dark brown.

***Head and thorax*.** Vertex (Figs 5C, 6A) shorter in middle line than wide at base (1: 1.30), width at apex narrower than at base (1: 1.31), middle part of anterior margin convex, lateral margins widened towards the end, lateral and submedian carinae distinct, Y-shaped carina indistinct. Frons (Figs 5E, 6B) longer in middle line than wide at widest portion (about 2.06: 1), widest at apex, median carina simple. Base of postclypeus (Figs 5E, 6B) as wide as apex of frons. Antennae (Figs 5E, 6B) with first segment longer than wide, shorter than second segment (1: 2.41). Pronotum (Figs 5C, 6A) nearly equal in length to vertex in midline (1.10: 1). Mesonotum (Figs 5C, 6A) longer than 1.40 times pronotum and vertex combined. Forewings (Fig. 5F) slender, longer than maximal width (3.41: 1).

***Male genitalia*.** Pygofer ventral margin longer than dorsal margin in lateral view (Fig. 6D), ventral margin slightly concave, posterior margin convex medially, in posterior view (Fig. 6C) with opening longer than wide, oval, ventral margin with a relatively short medioventral process, forked medially. Anal segment (Fig. 6C–F) ring-like, with a thick and long anal process at left lateroapical angle, taper the end, distinctly divided into 3 processes at apex. Genital styles (Fig. 6C, D, H–I) short, hogged, apical part pointed, curved inward. Aedeagus (Fig. 6J) with phallobase, phallus tubular, basal part thick, curved ventrally in the middle, basal and middle parts each with a dentate process, apical part with two unciform spinous processes; gonopore located at apex of phallus; phallobase slender and long, curved at apex.

#### Host plant.

Bamboo.

#### Distribution.

China (Tibet Province).

#### Etymology.

The species name is derived from the Latin word “*striola*”, referring to forewing with a dark brown stripe.

## ﻿Discussion

Host plant information is rarely recorded in Fulgoroidea and even in Delphacidae; most of the host information is recorded in Tropidocephalini. Species of the Tropidocephalini feed on Poaceae, with most reported plant associations involving bamboo. Many of these species are important or potential pests of bamboo (Chen 2003; Ding 2006; Chen and Tsai 2009). In *Bambusiphaga*, all species are known to exclusively feed on bamboo (Bambusoideae), of which *B.luodianensis* Ding, 1982, *B.citricolorata* Huang & Tian, 1979, *B.furca* Huang & Ding, 1979, *B.taiwanensis* (Muir, 1917) were reported to be one of the main stinging pests on bamboo (Yang et al. 1999; Liu and Chen 2008; Li et al. 2010; Hou and Chen 2013), occurring in 3~5 generations every year. It feeds on the tender parts of plants and has a significant impact on bamboo growth, making it one of the most important pests in bamboo forest production.

Based on data from published information and our field surveys, all species of *Bambusiphaga* were known from the Oriental region, and are especially species-rich in China, where 31 species are now recorded. However, at present, the genus is mainly distributed in Central China, East China, South China and Southwest China, and most species are known only from their type locality. Only 10 species have been reported outside their type locality and we believe that the actual distribution range of most species remains unclear. Therefore, further collection and investigation remain necessary to identify other undiscovered species and populations to better understand their ecological impacts and enhance the taxonomy of the group.

## Supplementary Material

XML Treatment for Bambusiphaga

XML Treatment for Bambusiphaga caudospina

XML Treatment for Bambusiphaga laterospina

XML Treatment for Bambusiphaga striola

## ﻿Additional information

### Author ORCIDs

Sha-Sha Lv https://orcid.org/0000-0001-5353-5082

Hong-Xing Li https://orcid.org/0000-0002-6427-8875

Lin Yang https://orcid.org/0000-0002-7841-5156

Yu-Bo Zhang https://orcid.org/0000-0002-6118-6190

Xiang-Sheng Chen https://orcid.org/0000-0001-9801-0343

### Data availability

All of the data that support the findings of this study are available in the main text.
